# Comparative analysis of clinical profile, laboratory profile and outcome in COVID-19 patients with and without hypothyroidism

**DOI:** 10.4314/gmj.v58i3.3

**Published:** 2024-09

**Authors:** B Sindhu Malini, Yoganathan Chidambaram, C P Clement Jenil Dhas, B K Navinkumar, S Sujith Kumar

**Affiliations:** Department of General Medicine, PSG Institute of Medical Sciences and Research, Coimbatore, Tamil Nadu, India

**Keywords:** COVID-19, hypothyroidism, outcomes, mortality, comorbidity

## Abstract

**Objectives:**

Previous studies suggest that patients' thyroid status might directly impact the course of Coronavirus disease 2019 (COVID-19). The objective of the study was to determine the clinical profile of COVID-19 patients with hypothyroidism and compare it with that of COVID-19 patients without hypothyroidism.

**Design:**

Retrospective observational study

**Setting:**

The study was conducted in a tertiary healthcare centre in Tamil Nadu between May and June 2021.

**Participants:**

The study included 117 patients admitted with hypothyroidism and COVID-19 as well as 117 age and Gender matched COVID-19 patients without hypothyroidism.

**Main outcome measures:**

Data regarding the demography, comorbidities, presenting symptoms, method of diagnosis of COVID-19, computed tomography (CT) severity score, Interleukin 6 (IL-6), D-dimer, oxygen requirement, number of days in hospital and outcome were collected for both groups. Data analysis was conducted, and p<0.05 was considered statistically significant.

**Results:**

The study comprised 234 patients over two months, from May to June 2021. Distribution of presenting symptoms showed that the hypothyroidism group presented with a higher incidence of fever (66.67%), loose stool (18.80%) and myalgia (7.69%). Results show that RTPCR+, O_2_ Requirement, death, D-dimer, IL-6, number of days admitted as well as CT-severity did not show any statistically significant differences (p>0.05) between both groups. The outcomes also showed that both groups reported four mortalities.

**Conclusions:**

The results of the study help conclude that the hypothyroidism status of a COVID-19 patient is not associated with higher severity of clinical symptoms, deranged laboratory values as well as mortality.

**Funding:**

None declared

## Introduction

The entire world had stood still as the Coronavirus disease 2019 (COVID-19) pandemic was declared a public health emergency on the 30^th^ of January 2020. Symptoms related to the COVID-19 disease were found to be fatal and resulted in many deaths worldwide.^1^ However, evidence suggested that the incidence of the disease was more common in people with comorbidities, thereby increasing the chance of mortality as COVID-19 infection can put an incredible strain on the different organ systems of an individual.^1^

The most common underlying comorbidities associated with COVID-19 were chronic diseases, such as cardiovascular diseases (CVD), hypertension, diabetes, and obesity.

A large population of patients who had recently recovered from Severe Acute Respiratory Syndrome Coronavirus 2 (SARS-CoV-2) infection were found to present with cardiac involvement (78%) and ongoing myocardial inflammation (60%), irrespective of past comorbidities or course of COVID-19 infection.^2^,^3^

Alongside these comorbidities, clinical studies also revealed cases of viral infection-related thyrotoxicosis after SARS-CoV-2 infection. However, the incidence of this condition is relatively low.^4^-^6^ Patient TSH levels fell to the hypothalamic reference range, similar to the clinical course of a thyrotoxic condition.^7^ This necessitated further research as physicians should be made aware of a potential association between COVID-19 and hypothyroidism.

This connection can also be seen concerning how the virus affects the hypothalamic-pituitary-thyroid axis.^7^

The relationship between thyroid hormone and immunological regulation has been widely studied in physiological and pathological contexts.^3^ However, evidence is required to emphasise the relationship between thyroid hormone and COVID-19 disease progression since immune system regulation affects how rapidly patients with COVID-19 develop the disease.^8^ Evidence related to how thyroid disorders affect the course of COVID-19 infection and disease is scarce. Particularly, conflicting findings have emerged regarding the prevalence of hypo- and hyperthyroidism among COVID-19 patients as well as the influence these multi-morbidities have on mortality in COVID-19 patients.^9^

In light of these considerations, the current investigation set out to evaluate the COVID-19 clinical profile in individuals who had previously received a diagnosis of underlying hypothyroidism to determine whether hypothy-roidism stands alone as a risk factor for COVID-19. The study's objectives were to study the clinical profile and outcome of COVID-19 patients with hypothyroidism and compare the clinical profile and outcome of COVID-19 patients with and without hypothyroidism.

## Methods

The retrospective observational study was conducted in a tertiary healthcare centre in Coimbatore, Tamil Nadu, India. All patients admitted with hypothyroidism and COVID-19 between May 2021 and June 2021 were included in the study. Age and gender-matched COVID-19 patients without hypothyroidism were also included in the study to facilitate comparison with the study group. The study was conducted after obtaining clearance from the Institutional Human Ethics Committee (PSG/IHEC/2023/Appr/Exp/139).

Patients were included if they were inpatients above the age of 18 diagnosed with COVID-19 and hypothyroidism. Age and gender-matched COVID-19 patients without hypothyroidism were also included as a comparison group. Patients whose records were not available or incomplete, as well as patients who were discharged against medical advice, were excluded from the study.

During the study period, 3910 adults suffering from COVID-19 were admitted. One hundred and twenty-two patients had hypothyroidism. After applying inclusion and exclusion criteria, 117 were included in the study. Gender and age-matched controls were selected from the consecutive patients to form the comparison group.

Data related to demography, comorbidities, presenting symptoms, method of diagnosis of COVID-19 (either RT-PCR or radiological), CT severity score, IL-6 (pg/ml), D-dimer (mg/L FEU), oxygen requirement, number of days in hospital and outcome (discharge or death) were collected for both cases and controls.

The type of D dimer measurement method used in the study was immunoturbidimetric assay using Innovance D dimer (by Siemens); quality control was done using Innovance D dimer controls, which are used for the assessment of precision and analytical bias in the normal and pathological range. IL-6 was measured using Chemiluminescent Immunoassay using Elecsys IL-6 (by Roche diagnostics), while quality control was done using Precicontrol Multimarket. The severity of lung involvement was assessed by using a 25-point CT severity score wherein Three lung lobes on the right and two lobes on the left were individually assessed, and percentage involvement of the lobe was noted based on visual assessment. Visual severity scoring of CT chest was classified as score-1 (<5% area involved), score-2 (5–25% area involved), score-3 (25–50% area involved), score-4 (50–75% area involved), score-5 (>75% area involved), making the total score 25. A CT severity score was assigned out of 25 based on the percentage area involved in each of the 5 lobes. The total CT score is measured by the sum of the individual lobar scores and ranges from 0 (no involvement) to 25 (maximum involvement) when all five lobes show more than 75% involvement.

### Statistical Analysis

The data were analysed using Microsoft Excel and Statistical Product and Service Solutions (SPSS software v. 28.0). Mean, mode and standard deviation were calculated for all quantitative measurements. The comparison of categorical variables between groups was analysed using two-sample proportion tests, and the comparison of continuous variables between groups was done using an independent sample t-test. A p-value <0.05 was considered statistically significant when comparing variables between both groups.

## Results

The present study comprised 234 patients, which included patients with hypothyroidism and without hypothyroidism, studied for two months from May to June. The mean age of the participants was 54±16 years. Table 1 depicts the distribution of gender within the two treatment groups. Most individuals in both the No-hypothyroidism and hypothyroidism groups are female. The distribution of comorbidities among the “non-hypothyroidism” group showed that the most common comorbidity among individuals was Diabetes (33.33%), followed by Hypertension (23.08%).

**Table 1:** Distribution of participants based on comorbidities

		Treatment	
		Non-Hypothyroid	Hypothyroid
**Gender**	Female	98 (83.80%)	16 (82.10%)
	Male	19 (16.20%)	21 (17.90%)
**SHT**	No	90 (76.92%)	88 (75.21%)
	Yes	27 (23.08%)	29 (24.79%)
**CAD**	No	113 (96.58%)	107 (91.45%)
	Yes	4 (3.42%)	10 (8.55%)
**DLP**	No	116 (99.15%)	112 (95.73%)
	Yes	1 (0.85%)	5 (4.27%)
**CVA**	No	117 (100%)	117 (100%)
	Yes	0 (0%)	0 (0%)
**CKD**	No	117 (100%)	114 (97.44%)
	Yes	0 (0%)	3(2.56%)
**CLD**	No	115 (98.29%)	117 (100%)
	Yes	2 (1.71%)	0 (0%)
**COPD/ASTHAM**	No	113 (96.58%)	115 (98.29%)
	Yes	4 (3.42%)	2 (1.71%)
**DIABETES**	No	78 (66.67%)	70 (59.83%)
	Yes	39 (33.33%)	47 (40.17%)

Note: SHT: Systematic Hypertension, CAD: Coronary Artery Disease, DLP: Dyslipidemia, CKD: Chronic Kidney Disease, CVA: Cerebrovascular Accident, COPD: Chronic Obstructive Pulmonary Disease, CLD: Chronic Liver Disease

A small portion of individuals suffered from coronary artery disease (CAD) (3.42%), COPD/Asthma (3.42%), Chronic Liver Disease (CLD) (1.71%) and Dyslipidemia (0.85%).

Among the “Hypothyroidism” group, the most common comorbidity reported was Diabetes (40.17%), followed by Hypertension (24.79%). Other comorbidities observed included CAD (8.55%), Dyslipidemia (4.27%), COPD/asthma (1.71%), and CKD (2.56%).

From Table 2, the distribution of the presenting symptoms was assessed. The results show that the hypothyroidism group presented with a higher incidence of fever (66.67%), loose stool (18.80%), myalgia (7.69%) and fatigue (5.13%). In contrast, in the non-hypothyroidism group, the most common symptoms observed were cough (63.25%) and dyspnea (23.93%). The non-hypothyroidism group also had 2.71% of asymptomatic patients, while none of the patients from the hypothyroidism group were asymptomatic.

**Table 2:** Distribution of patients based on presenting symptoms

		Treatment	
		Non-Hypothyroid	Hypothyroid
**Fever**	No	43(36.75%)	39 (33.33%)
	Yes	74 (63.25%)	78 (66.67%)
**Cough**	No	43 (36.75%)	46(39.32%)
	Yes	74 (63.25%)	71(60.68%)
**Dyspnea**	No	89 (76.07%)	94 (80.34%)
	Yes	28 (23.93%)	23 (19.66%)
**Loose Stool**	No	105 (89.74%)	95 (81.20%)
	Yes	12 (10.26%)	22 (18.80%)
**Myalgia**	No	110 (94.02%)	108 (92.31%)
	Yes	7 (5.98%)	9 (7.69%)
**Fatigue**	No	114 (97.44%)	111 (94.87%)
	Yes	3 (2.56%)	6(5.13%)
**Asymptomatic**	No	115 (98.29%)	117 (100%)
	Yes	2 (2.71%)	0 (0%)

Table 3 presented data related to the comparison of categorical variables between both groups and showed that variables such as RTPCR+ (p=0.7956), O_2_ Requirement (p=0.7072) and death (both groups reported 4 deaths) did not show any statistically significant differences. The comparison of qualitative variables between both groups showed that variables such as D-dimer (p=0.916), IL-6 (p=0.103), number of days admitted (p=0.511) as well, and CT-severity (p=0.271) did not show any statistically significant differences.

**Table 3:** Comparison of categorical and quantitative variables between two groups

Categorical variables					
Variables		Treatment		Total	p-value
		Non-Hypothyroid	Hypothyroid		
**RTPCR+**	**Yes**	108 (92.31%)	110 (94.02%)	218 (93.16%)	0.7956
	**No**	9 (7.69%)	7 (5.98%)	16 (6.84%)	
**Total**		117	117	234	
**O**_**2**_ **Requirement**	**Yes**	15 (12.82%)	18 (15.38%)	33 (14.10%)	0.7072
	**No**	102 (87.18%)	99 (84.62%)	201 (85.90%)	
**Total**		117	117	234	
**Death**	**Yes**	4 (3.42%)	4(3.42%)	8 (3.42%)	
	**No**	113 (96.58%)	113(96.58%)	226 (96.58%)	
**Total**		117	117	234	
**Quantitative variables**					
**Variables**	**Treatment**	**Mean**	**Std. Deviation**	**Std. Error**	**p-value**
**D Dimer**	**Non-Hypothyroid**	1.9849	4.5991	0.4252	0.916
	**Hypothyroid**	2.0477	4.5305	0.4188	
**IL-6**	**Non-Hypothyroid**	30.4953	36.5299	3.3772	0.103
	**Hypothyroid**	47.3515	105.0327	9.7103	
**No. Days in Hospital**	**Non-Hypothyroid**	7.22	4.765	0.441	0.511
	**Hypothyroid**	6.86	3.479	0.322	
**CT-Severity**	**Non-Hypothyroid**	5.70	5.120	0.473	0.271
	**Hypothyroid**	6.46	5.418	0.501	

Note: p<0.05: statistically significant

## Discussion

The thyroid gland and the COVID-19 virus infection are engaged in complex interplay via hormones and immunomodulatory signalling molecules; these connections have been established in physiological and pathological settings.^10^ Studies have reported that, to a lesser extent, hypothyroidism could negatively impact the outcome of COVID-19 infection, resulting in increased mortality.^11^,^12^,^13^ Since the hypothalamus and pituitary, which control the operation of most endocrine glands, both express angiotensin-converting enzyme 2 (ACE2), the primary protein to which SARS-CoV-2 attaches to enter host cells, it is not surprising that SARS-CoV-2 can affect the endocrine system. The thyroid gland, the hypothalamus, and the pituitary also express ACE2 and may be directly impacted by COVID-19. Assuming that hypothyroidism leads to immune system dysfunctions and that ACE2 is expressed in thyroid gland, one could speculate that hypothyroidism might impact the outcomes in COVID-19 patients.^14^ Mechanisms that can cause this include an indirect effect of systemic inflammatory immune response, dysfunction of the hypothalamic-pituitary-thyroid (HPT) axis leading to decreased serum TSH or a direct effect of SARS-CoV-2 on target cells.^15^

The present study comprised of patients with hypothyroidism and without hypothyroidism studied for two months from May to June, and the mean age of the participants was 54±16 years. A study by Burekovic A et al. showed a similar age profile with a mean age of 55±4 years, which is in complete agreement with this study.^16^ The results of a study conducted by Lui DTW et al. to assess thyroid dysfunction to immune profile, disease status and outcome in patients with COVID-19 showed that the mean age of the participants was 53.5±17.2 years.^17^ The results reported the gender distribution between the two groups and showed that it included more female participants than males. Conversely, the proportion of males is lower in both groups but slightly higher in the hypothyroidism group (17.9% vs 16.2%). A previously conducted study showed that 53 of 58 participants were female, which completely agrees with this study.^16^ An increased number of female participants among hypothyroidism patients was also observed in a study by Bogojevic M et al.^18^ Another previously conducted study showed a slightly higher number of male participants. This contrasted with the results of the present study.^17^ When we consider the individual conditions assessed here, studies on gender disparities concerning COVID-19 did not show female predisposition.^19^ However, autoimmune hypothyroidism showed a fourfold predisposition toward female participants.^20^

The distribution of comorbidities among the group with hypothyroidism showed that most of them suffered from diabetes, followed by hypertension and CAD, while none of them suffered from CVA or CLD. The group without hypothyroidism also showed similar results. A previously conducted study showed that hypertension was reported by 27.2% of patients and 13.1% of participants suffered from diabetes. This was in contrast with the results of the present study, wherein diabetes was the most comorbidity, followed by hypertension among individuals with hypothyroidism.^17^ Another previously conducted study showed a similar difference, with hypertension being the most common comorbidity, followed by diabetes.^18^ A Brazilian study reported that coronary (p=0.015) and CKD (p=0.001) incidence was higher in patients with hypothyroidism, and this is in stark contrast to the results of the present study.^21^ The results are expected as hypertension and diabetes are the most common comorbidities seen among most populations.^22^ The outcome can also be explained by the fact that India is known as the diabetes capital of the world, with 69.9 million people expected to have diabetes by 2025 and 80 million by 2030. This indicates that a 266% growth is anticipated in the developing nation.^23^

The distribution of the presenting symptoms was assessed, and the results show that the hypothyroidism group presented with a higher incidence of fever, loose stool, myalgia, and fatigue. In contrast, the non-hypothyroidism group presented with cough and dyspnea. The non-hypothyroidism group also had two asymptomatic patients, while none of the patients from the hypothyroidism group were asymptomatic. Fever was the most commonly observed symptom among the participants with hypothyroidism, followed by cough, and this was similar to the results of a study by Lui DTW et al., which reported that fever was the most common symptom among COVID-19 patients with hypothyroidism (66.7%) followed by cough (58.3%).^17^ However, fever and cough were also the most common symptoms in the non-hypothyroid group, and the groups were comparable in this regard. This can be explained by fever being the human body's reaction to fight off infections, especially COVID-19. This makes further research into the symptoms of hypothyroidism in COVID-19 patients essential.

Comparison of variables between both groups showed that variables such as RTPCR+ (p=0.7956), O_2_ Requirement (p=0.7072), D-dimer (p=0.916), IL-6 (p=0.103), number of days admitted (p=0.511) as well as CT-severity (p=0.271) did not show any statistically significant differences. The incidence of death was similar in both groups. D-dimer levels were reported to be lower among hypothyroidism patients in a previously conducted study, and this does not follow the results of the present study.^21^ A study conducted by van Gerwen M et al. concluded that hypothyroidism is not associated with an increased risk of COVID-19-related hospitalisation or a worse outcome, including death, and this was per the results of the present study.^24^ A similar result was obtained by a study conducted in Iran wherein patients with hypothyroidism did not show any difference in mortality when compared to patients without hypothyroidism.^25^,^26^ Another study also showed that length of stay and mortality among COVID-19 patients were not associated with hypothyroidism status if participants.^18^ Another study showed that thyroid dysfunction had no effect on COVID-19 susceptibility and severity.^27^

The limitations of our study were the single-centre design and lack of a sample size estimation, which could have affected the generalizability of the study results. The study design could have been more robust by including participants from other hospitals or regions and estimating a sample size, thereby eliminating bias. The clinical implications of the present study are that comorbidities, clinical symptoms and treatment variables seen in hypothyroid patients with COVID-19 disease are comparable to those reported among COVID-19 patients without hypothyroidism. Hence, COVID-19 disease, along with hypothyroidism, is not associated with any changes in study variables, thus making the treatment of these patients a simpler task as there is no fear of lower thyroid levels affecting prognosis. The results of the present study can be built upon due to the heterogeneous level of evidence and results, thus necessitating the need for a higher level of evidence concerning the effects of thyroid function in COVID-19 patients.

## Conclusion

The results of the study help conclude that hypothyroidism disease in COVID-19 patients is not associated with any form of adverse outcomes, increase in disease severity and mortality rates. The results of the present study can be used to assign necessary treatment to COVID-19 patients irrespective of hypothyroidism status.
